# Amygdala reactivity and ventromedial prefrontal cortex coupling in the processing of emotional face stimuli in attention-deficit/hyperactivity disorder

**DOI:** 10.1007/s00787-021-01809-3

**Published:** 2021-06-13

**Authors:** Tammo Viering, Jilly Naaijen, Daan van Rooij, Christiane Thiel, Alexandra Philipsen, Andrea Dietrich, Barbara Franke, Jan Buitelaar, Pieter J. Hoekstra

**Affiliations:** 1 Biological Psychology, Department of Psychology, School of Medicine and Health Sciences, Carl-Von-Ossietzky Universität Oldenburg, Postfach 2503, 26111 Oldenburg, Germany; 2 Department of Child and Adolescent Psychiatry, University Medical Center Groningen, University of Groningen, Groningen, The Netherlands; 3 Department of Cognitive Neuroscience, Radboud University Medical Center, Donders Institute for Brain, Cognition and Behaviour, Nijmegen, The Netherlands; 4 Centre for Cognitive Neuroimaging, Radboud University, Donders Institute for Brain, Cognition and Behaviour, Nijmegen, The Netherlands; 5 Research Center Neurosensory Science, Carl-Von-Ossietzky Universität Oldenburg, Oldenburg, Germany; 6 Cluster of Excellence “Hearing4all”, Carl-Von-Ossietzky Universität Oldenburg, Oldenburg, Germany; 7 Department of Psychiatry and Psychotherapy, University of Bonn, Bonn, Germany; 8 Department of Human Genetics, Radboud University Medical Center, Donders Institute for Brain, Cognition and Behaviour, Nijmegen, The Netherlands; 9 Department of Psychiatry, Radboud University Medical Center, Donders Institute for Brain, Cognition and Behaviour, Nijmegen, The Netherlands; 10 Karakter Child and Adolescent Psychiatry University Centre, Nijmegen, The Netherlands

**Keywords:** ADHD, Emotion recognition, Emotion dysregulation, Task fMRI, Amygdala, Prefrontal cortex

## Abstract

Impaired emotion recognition is common in individuals with attention-deficit/hyperactivity disorder (ADHD) and may, via deficient emotion self-regulation, relate to the frequently co-occurring affective and social problems. The present study used an emotional face-matching task and functional magnetic resonance imaging (fMRI) to investigate neural responses during the processing of angry and fearful faces and visuo-spatial control stimuli. Additionally, measures for emotion dysregulation, ADHD type, and age were investigated in relation to the behavioral and neural fMRI data. We utilized a sample of 61 adolescents/young adults with ADHD and 51 age-matched healthy controls (age range: 12–28 years). Participants with ADHD had higher emotion dysregulation scores than controls. They also reacted slower and less accurate in response to emotional but not visuo-spatial control stimuli. Neural response differences between emotional and visuo-spatial trials were significantly smaller in cases, particularly in the left amygdala. While coupling between the right amygdala and bilateral ventromedial prefrontal cortex was stronger for emotional than visuo-spatial stimuli in control subjects, levels of positive coupling between the trial types did not significantly differ in participants with ADHD. Neither emotion dysregulation scores, nor ADHD type or age were related to the behavioral and neural processing alterations during the emotional face-matching task. Results indicate that emotion recognition deficits in ADHD are particularly associated with lower amygdala activation to emotional stimuli and alterations in the functional connections of the amygdala to medial prefrontal areas. Emotion recognition deficits and associated neural alterations were unrelated to emotion dysregulation, ADHD type, or age.

## Introduction

With an estimated prevalence rate of 5.3% in children and adolescents [1] and 2.5% in adults [2], attention-deficit/hyperactivity disorder (ADHD) is one of the most common neurodevelopmental disorders. Besides core symptoms of inattention and hyperactivity/impulsivity, emotion dysregulation is a frequently reported problem [3, 4]. Emotion dysregulation refers to the inability to appropriately modulate emotional responses [5] and is present in as many as 25–45% of children and 30–70% of adults with ADHD [6]. Its co-occurrence in ADHD is associated with worse clinical outcome, risky behavior, and social impairments [6–8].

An important aspect of adequate emotion regulation is the accurate recognition and interpretation of emotional stimuli. Individuals with ADHD were found to be less accurate in identifying emotions. Besides other factors, such as subconscious and experience based reward estimations or cognitive reappraisal [9, 10], emotion dysregulation in ADHD may be related to emotion recognition deficits [6], as both require the ability to direct attention toward or away from emotional stimuli. Some of the inappropriate emotional behavior may be attributed to emotion recognition deficits [11–14].

While most neuroimaging research in ADHD has focused on frontostriatal, frontocerebellar, and frontoparietal circuits, few studies investigated the functional connections between the amygdala and the prefrontal cortex during tasks requiring emotion recognition in ADHD. The limited number of studies regarding emotion perception and recognition/matching during functional magnetic resonance imaging (fMRI) revealed evidence for case–control differences in affective arousal structures, including the ventral striatum, cingulate cortex, anterior insula, and, most consistently, (left) amygdala. Significant results have been repeatedly shown for the amygdala, although with inconsistency regarding the laterality or direction of the effects [15–20]. Resting state fMRI and anatomical MRI research in ADHD suggest altered connectivity in related structures (anterior default mode network, ventromedial prefrontal cortex (vmPFC), orbitofrontal cortex, and insula), which have been frequently associated with emotion recognition and regulation [21–25]. Indeed, evidence for a link between amygdala—prefrontal cortex coupling and emotion recognition has been presented in healthy individuals [26, 27]. Together with the well-established association between frontostriatal network anomalies and ADHD, particularly with impulsivity-hyperactivity symptoms [3, 28], this suggests that the functional connections between amygdala and prefrontal cortex may be related to emotion recognition deficits in ADHD. These, in turn, may ultimately contribute to the frequent emotion dysregulation problems seen in ADHD. However, it must be emphasized that the mentioned structures and circuits take over tasks beyond the correct recognition of emotions. The frontostriatal networks, in particular, are essential for reward estimation, decision-making, emotion processing, and emotion regulation [9, 29].

The main objective of the present study was to investigate amygdala reactivity and functional connections of the amygdala and prefrontal cortex in adolescents and young adults with ADHD as compared to healthy controls during the processing of fearful and angry facial stimuli. We chose matching of fear and anger given the previously reported conduct problems and impairments to recognize these emotions in children with ADHD [30]. A secondary objective was to investigate whether amygdala reactivity or alterations of the fronto-amygdala axis were associated with emotion dysregulation, as measured by the emotional lability subscale of the Conners’ parent rating scale, with ADHD type, or age. The study utilized data of the NeuroIMAGE study [31] with a well-established fMRI emotional face-matching task [17, 32, 33]. We hypothesized that individuals with ADHD would show longer reaction times and worse accuracy than controls during the emotional trials of the task. Further, we expected that this behavioral pattern would be accompanied by divergent amygdala activation and altered functional connectivity between the amygdala and prefrontal structures.

## Methods and material

### Participants and procedures

Individuals with ADHD and healthy controls participated in NeuroIMAGE II, the third wave of an integrated genetics-cognition-MRI-phenotype project focusing on ADHD [31]. Initial inclusion criteria for first-wave participants with ADHD were a combined type ADHD diagnosis, availability of one or more siblings, age between 6 and 18 years, and availability of the participant, sibling, and at least one biological parent for DNA collection. Exclusion criteria applying to all participants were IQ < 70, inability to understand study procedures, diagnoses of autism or schizophrenia, and neurological disorders. For controls, it was additionally required that neither they, nor any of their first-degree relatives, had a prior ADHD diagnosis. The current wave took place 9 years after the first wave.

The diagnostic procedure was based on DSM-IV-TR criteria [34] and is described in more detail by van Rhein et al. [31]. Clinical ADHD diagnoses conferred by experienced clinicians were confirmed by combining information from a semi-structured interview (Kiddie Schedule for Affective Disorders (K-SADS [35])) and parent, teacher, and self-report versions of the Conners’ rating scale (CPRS-R:L [36], CTRS-R:L [37] & CAARS-R:L [38]). Emotion lability scores (as an index for emotion dysregulation) were derived from the parent-rated CPRS-R:L. The emotion lability subscale consists of three items (i.e., unpredictable mood changes, temper tantrums, and tearfulness) and has been utilized repeatedly to assess emotion lability in ADHD [39]. All data presented in this study, including the diagnostic questionnaires and emotional lability scores, refer to the present wave of the project and were collected on the same day as MRI scanning.

The group examined here is a subsample of the 302 participants included in the third wave. The total group is composed of individuals who took part in the previous waves and new recruits added on account of dropouts (particularly within the control group). For the present study, however, only those individuals who could unambiguously be assigned to either the control or ADHD group were considered. These ambiguous or subthreshold participants had a symptom count that was neither indicative of an ADHD diagnosis (≤ 6 for children, ≤ 5 for adults) nor classified them as unaffected (≥ 3 for children, ≥ 2 for adults). Also, individuals who did not meet the criteria for daily living impairments or onset-age but had multiple symptoms fell into this category (for more details see von Rhein et al. [31]). From this sample of 145 participants who also had available fMRI data, a number of participants were removed prior to analyses due to left-handedness (*n* = 21, only right handed participants were studied to reduce variability due to lateralization differences), low quality of behavioral (participants whose emotional face-matching accuracy was more than three standard deviations worse than the group average; *n* = 3), imaging data (*n* = 6, exclusion limit of 3 mm on rotational and translational movement), and incomplete diagnostic data (*n* = 3). This resulted in a final sample of 112 individuals (*n* = 61 ADHD, *n* = 51 healthy controls, similar in age (12–28 years) and sex). Among the 61 participants with ADHD, 36 had predominantly inattentive ADHD, 6 predominantly hyperactive/impulsive ADHD, and 19 combined ADHD. Due to the small number of participants with hyperactive/impulsive ADHD, they were merged with participants with a combined diagnosis in all analyses.

Stimulant medication was discontinued 48 hours prior to testing. Data acquisition took place at the Donders Institute for Cognitive Neuroimaging, Radboud University Nijmegen, Netherlands. Participants (and their parents when < 18 years old) gave written informed consent for participation. Ethical approval was granted by the Regional Ethics Board (Centrale Commissie Mensgebonden Onderzoek: CMO Regio Arnhem Nijmegen, ABR: NL41950.091.12).

### Neuropsychological task during fMRI

**Fig. 1:**
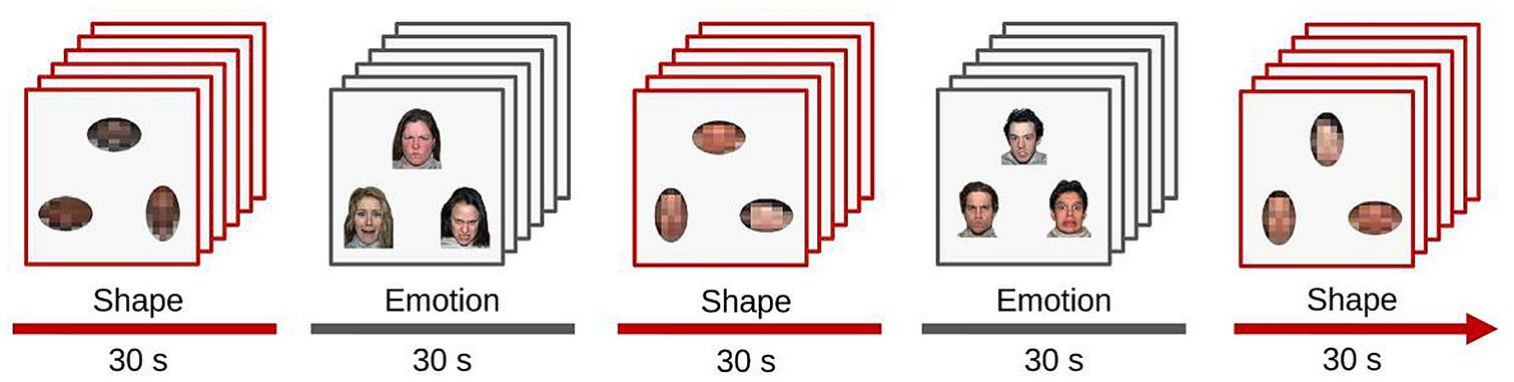
Illustration of the emotional face-matching task applied during fMRI scanning

In two emotion matching and three visuo-spatial control blocks, each with six trials of 5 s in length, participants were asked to match the facial emotion (fear or anger) or spatial orientation (vertical or horizontal ellipses) of an upper stimulus with one of two lower stimuli, in line with a previous study (31). The three simultaneously presented facial stimuli always depicted faces of different individuals of the same sex (taken from http://www.macbrain.org). Half the trials depicted women and the other half men. Ellipses of the visuo-spatial trials consisted of scrambled face-stimuli pixels. Responses were given with left or right button presses. The task is well-established and served to investigate drug effects on amygdala reactivity [17, 32, 33]. Instead of neutral facial expressions, geometric shapes were used as control stimuli, since the former could be perceived ambiguously and could cause unwanted amygdala reactivity [33, 40]. Figure 1 summarizes the applied task.

### fMRI data acquisition

Functional MRI data acquisition was performed with a 1.5 T Magnetom Avanto (Siemens AG, Erlangen, Germany). To acquire T2*-weighted blood oxygen level dependent (BOLD) images, accelerated multi-echo EPI sequences, which additionally reduce image distortion and increase BOLD sensitivity, were used (TR = 2660 ms, TE1/TE2/TE3/TE4/TE5 = 7.7/17.3/27.0/37.0/46.0 ms). For each volume, 37 axial slices were generated in an interleaved and ascending order (flip angle = 90°, FoV = 224 × 224 mm^2^, voxel- size = 3.5 × 3.5 × 3.0 mm^3^, inter-slice gap = 0.5 mm, GRAPPA 2). Echo-time (TE) weighted summation was used to combine all five echoes into a single data set. T1-weighted high-resolution structural volumes were acquired with an MPRAGE sequence (TR = 2730 ms, TE = 2.95 ms, TI = 900 ms, flip angle = 9°, FoV = 256 × 256 mm^2^, voxel- size = 1.0 × 1.0 × 1.0 mm^3^, GRAPPA 2).

### Behavioral data analysis

Reaction time and hit rate differences between the control and ADHD group during the emotion matching task trials were analyzed using mixed linear models with diagnostic status as the grouping variable and task condition as the repeated measures variable (one model for reaction time and one for hit rate). Using partial sums of squares *F* tests we investigated the impact of the different model regressors. Emotion dysregulation, age, medication status, and sex were entered as additional covariates. As an intrinsic component of ADHD, IQ, with its typically strong relation with the diagnostic status, was not considered. In cases of significant interactions between diagnostic status and task condition, Bonferroni-corrected *t* tests were applied to investigate the diagnosis-specific differences within the different task conditions (corrected α = 0.0125). The statistical analysis of the behavioral data was conducted with R software [41].

### fMRI data analysis

FMRIB software library (FSL 5.0.11 [42]) was used for fMRI data processing. Functional images were skull stripped using FSL BET [43] and realigned to the middle volume of each time series to correct for head motion. Motion parameters for rotation and translation were calculated and the exclusion limit was set to 3 mm of absolute movement. Images were co-registered to the individual T1-weighted structural images. The volumes were spatially smoothed using a 6 mm full width at half maximum (FWHM) Gaussian kernel. To remove secondary motion artifacts, ICA-AROMA [44] was used. High-pass filtering was applied at 0.008 Hz and nuisance regression was used to remove residual noise of the white matter, cerebrospinal fluid (CSF), and linear signal drifts of overall brain activity. We used CSF and white matter masks obtained during a preceding segmentation of the T1-weighted structural scan. Prior to subject-level analysis, the preprocessed images were warped to MNI152 space (Montreal Neurological Institute, Montreal, Canada). During quality assessment and all conducted preprocessing steps of the individual fMRI datasets, researchers were unaware of group memberships.

At subject level, GLM were generated with FSL FEAT [45] to estimate statistical parametric maps. GLM consisted of four regressors modelling the emotion and visuo-spatial control blocks (length 30 s) and their respective temporal derivatives (Fig. 1). All regressors were convolved with the double-gamma hemodynamic response function (HRF) provided by FSL FEAT. The contrast of interest that was used for subsequent second level analyses contrasted the responses to blocks of angry or fearful face stimuli against the responses to blocks of horizontally or vertically oriented ellipses (emotion > shape). Due to the briefness of the fMRI task and the high frequency of correct responses, an event-related model that differentiates between false and correct trials or the gender of the respective stimuli could not be used.

With the individual beta contrast maps and associated maps of variance estimates, FSL FLAME 1 was used to calculate *z* value images for different contrasts and group-level models. Using the previously mentioned grouping algorithm, a two-group GLM with binary regressors for participants with ADHD and healthy controls was created (ADHD-HC GLM, *n* = 112). Age and sex were demeaned across participants and included as covariates of no interest. Since a somewhat lower IQ and use of medication is an intrinsic feature of ADHD [46], IQ and medication status were originally not added as covariates for the fMRI models. To ensure that these two variables did not influence any relevant results, we ran an additional model with these covariates and found no main or interaction effects. Consequently, they were not considered for the final model.

Statistical maps were calculated for the groups’ mean effects and the between group contrasts (ADHD > HC and ADHD < HC). A similar model, but with binary regressors for the groups with predominantly inattentive ADHD and combined or hyperactive/impulsive ADHD, was created to evaluate differences between ADHD participants with and without hyperactive/impulsive symptoms. To investigate possible age- and emotion dysregulation-dependent effects, models for the two main participant groups in interaction with these continuous covariates were used.

Region of interest (ROI) analyses were conducted with ROI masks of the left and right amygdala (240 and 280 voxels per mask; calculated from the Harvard–Oxford Atlas, thresholded at 50% and binarized; Fig. 3B). Hemisphere-specific masks were chosen because hemispheric differences in emotion processing are suspected [47] and previous studies have rarely found bilateral but rather mostly left-sided effects [15, 17, 18]. Mean beta values (averaged across all voxels within the ROI) were extracted from the individual emotion > shape contrasts. Similar to the behavioral analysis, mixed linear models were constructed. Diagnostic status and laterality, as repeated measure, were used as grouping variables, while the aforementioned mean beta values of the amygdala were added as dependent variable. Again, partial sums of squares *F* tests were conducted and the same variables that had been used for the behavioral analysis were considered as covariates to investigate additional main or interaction effects. Subsequent *t* tests were performed to investigate the hemisphere-specific group mean differences. The statistical analysis of the mean beta values was conducted with R software [41].

Further analyses used psychophysiological interaction (PPI) maps to investigate functional connectivity between BOLD responses in the left and right amygdala and other parts of the brain (amygdala seeds were taken from the Harvard–Oxford Atlas) [48]. PPI analyses identify areas whose activation levels depend on the interaction between a seed region and an experimental parameter. PPI estimate contextual connectivity changes between seed regions and other brain areas [48]. For the present analyses, the convolutions of the double-gamma HRF with the emotion > shape contrasts were chosen as experimental parameters. FSL FEAT with its standard procedures was used for implementation. The group-level analysis was conducted using the previously described two-group GLM. Following the PPI analyses, individual time series data of the amygdala and clusters, whose coupling with the amygdala was found to significantly depend on the trial condition, were extracted to calculate estimates for condition-specific amplitudes of covariance change. In other words, we calculated by how many multiples the average covariance between activity of the amygdala and any psychophysiologically interacting area changed from the visuo-spatial to the emotional condition. The alternative use of correlations for this purpose could be problematic for the interpretation of the results since a certain ambiguity arises with regard to shared and unshared signal components [49]. Results of the fMRI analyses are presented at a significance level of *p* < 0.01 and after cluster-level family-wise error (FWE) correction with cluster forming thresholds of 2.3 [50].

## Results

### Sample characteristics

Demographic details are provided in Table 1. Age and sex did not significantly differ between the ADHD and healthy control group. Participants with ADHD showed more ADHD symptom and higher emotion dysregulation scores. Compared to controls, participants with ADHD had a significantly lower IQ (still in the normal range for both groups), were more frequently diagnosed with oppositional defiant disorder (ODD) or conduct disorder (CD), and used stimulant medication more often. ADHD type did not significantly differ with age, sex, IQ, emotion dysregulation scores, and stimulant use.

**Table 1:** Sample characteristics of the healthy control and ADHD group as well as of presentation specific ADHD subgroups

		ADHD					
Group	HC	Total	Inattentive	Combined and hyperactive/impulsive	HC vs. ADHD group comparisons		
	*N* = 51	*N* = 61	*N* = 36	*N* = 25			
	Mean ± SD	Mean ± SD	Mean ± SD	Mean ± SD	Test statistic	*p* value	Effect-size
Age (years)	20.2 ± 3.2	20.0 ± 3.5	20.2 ± 3.7	19.70 ± 3.2	*T* = 0.2833	0.778	*d* = 0.053
IQ (WISC/WAIS)	114 ± 11.7	97 ± 18.1	98 ± 18.1	94.4 ± 18.3	*T* = 6.2708	< 0.001	*d* = 1.146
Emotional lability (CPRS-R:L)	43.9 ± 3.1	53.9 ± 13.2	53.83 ± 13.9	53.96 ± 12.4	*U* = 740.5	< 0.001	*d* = −0.524

	Median (range)	Median (range)	Median (range)	Median (range)			
DSM-IV ADHD, inattentive (K-SADS)	0 (0–7)	7 (4–9)	7 (5–9)	7 (4–9)	*U* = 31.5		*d* = −0.980
DSM-IV ADHD, inattentive (K-SADS)	0 (0–7)	7 (4–9)	7 (5–9)	7 (4–9)	*U* = 31.5	< 0.001	*d* = −0.980
DSM-IV ADHD, hyperactive/impulsive (K-SADS)	0 (0–2)	5 (0–9)	4 (0–9)	7 (6–9)	*U* = 130	< 0.001	*d* = −0.916
	*n*	*n*	*n*	*n*			
Sex (male)	29 (57%)	38 (62%)	22 (61%)	16 (64%)	*χ*^2^ = 0.152	0.696	*φ*_c_ = 0.001
Stimulant user (yes)	0 (0%)	22 (36%)	13 (36%)	9 (36%)	*χ*^2^ = 20.662	< 0.001	*φ*_c_ = 0.430
DSM-IV ODD (K-SADS)	0 (0%)	13 (21%)	7 (19%)	6 (24%)	*χ*^2^ = 28.310	< 0.001	*φ*_c_ = 0.503
DSM-IV CD (K-SADS)	0 (0%)	2 (3%)	1 (3%)	1 (4%)	*χ*^2^ = 9.180	0.010	*φ*_c_ = 0.286

Means between groups were compared with independent sample *t* tests or Mann–Whitney *U* tests. Frequency distributions were compared with Pearson’s chi-squared (*χ*^2^) test. For the CPRS-R:L *t* scores are presented, while for the K-SADS symptom counts are given

*N* number of participants, *n* number of participants within subgroups, *SD* standard deviation, *HC* healthy controls, *DSM-IV* diagnostic and statistical manual of mental disorders, 4th edition, *ADHD* attention-deficit/hyperactivity disorder *IQ* intelligence quotient, *K-SADS* Kiddie schedule for affective disorders and schizophrenia, *CPRS-R:L* Conners’ parent rating scale, revised, long version, *ODD* oppositional defiant disorder, *CD* conduct disorder

### Behavioral results

Two mixed linear models were constructed and partial *F* tests were used to investigate the ADHD- and task condition-specific influences on the behavioral results of the emotional face-matching task. Neither for reaction times nor for hit rates did the considered covariates show significant effects. Thus, no additional covariates were entered into the final models. Diagnostic status and task condition had a significant interaction effect on the average reaction times (F(1,110) = 20.814, *p* < 0.001, partial-η^2^ = 0.159). Post hoc comparisons between the two participant groups indicated significant mean difference in emotion and visuo-spatial trials (emotion trials: ADHD mean ± SD = 1.678 s ± 0.388 s, healthy control mean ± SD = 1.378 s ± 0.244 s, t(df = 103) = 4.946, *p* < 0.001, d = 0.9; visuo-spatial trials: ADHD mean ± SD = 0.913 s ± 0.166 s, healthy control mean ± SD = 0.838 s ± 0.223 s, t(df = 109) = 2.053, *p* < 0.001, d = 0.0.38). For mean hit rates, no significant interaction effect of the diagnostic status and task condition could be found. Solely a significant main effect of the diagnostic status could be detected (F(1,110) = 7.919, *p* = 0.006, partial-η^2^ = 0.159). Although not significant, the average mean difference in hit rates between ADHD and healthy control subjects was more evident in emotion trials than in visuo-spatial trials (emotion trials: ADHD mean ± SD = 0.915 s ± 0.092 s, healthy control mean ± SD = 0.958 s ± 0.063 s; visuo-spatial trials: ADHD mean ± SD = 0.926 s ± 0.074 s, healthy control mean ± SD = 0.942 s ± 0.066 s).

### fMRI results

#### Group comparison with whole-brain analyses

**Fig. 2:**
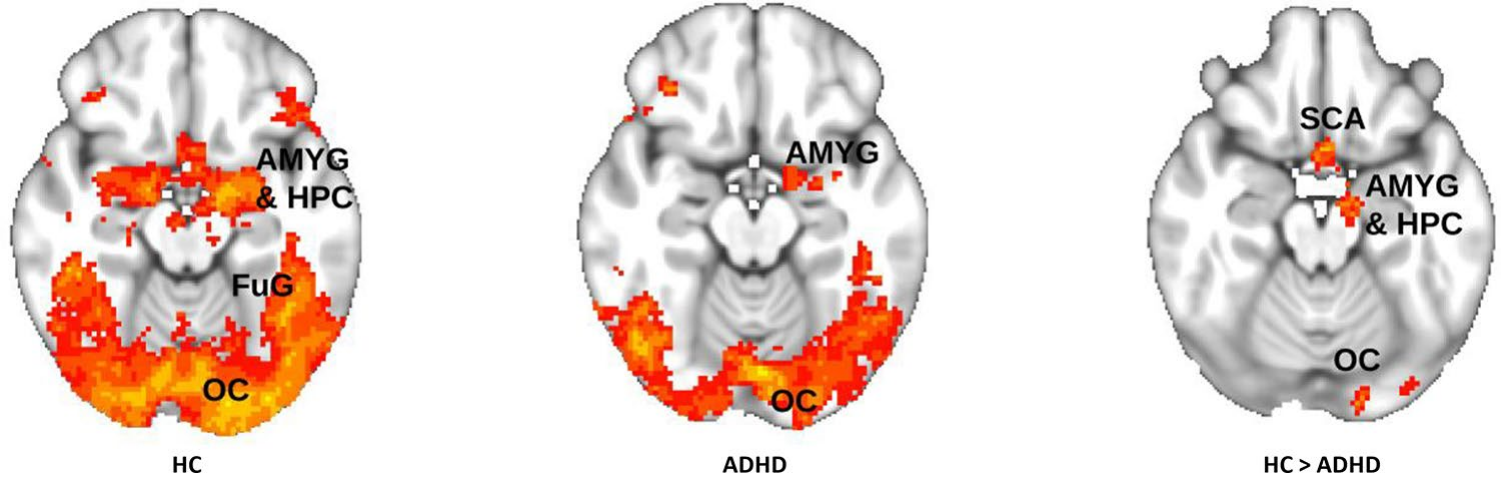
Neural activity for emotional faces versus a visuo-spatial control condition. Single group and HC > ADHD contrasts are displayed at Z = −16/−16/−19 (*p* < 0.05; FWE corrected; left/right reversed). *ADHD* attention-deficit/hyperactivity disorder, *AMYG* amygdala, *FuG* fusiform gyrus, *HC* healthy controls, *HPC* hippocampus, *OC* occipital cortex, *SCA* subcallosal area

To detect brain activity differences in group-specific BOLD activation during the emotion matching task (emotion > shape contrasts), the ADHD > HC and ADHD < HC contrasts of the ADHD-HC GLM were used. The ADHD > HC contrast did not reveal any significant clusters after FWE cluster-level correction. For the ADHD < HC contrast, significant clusters were present in the left amygdala, hippocampus, and subcallosal gyrus, cuneus and lingual gyrus, right superior and middle temporal, and left lateral occipital cortex and fusiform gyrus. Figure 2 shows neural activity in occipital and medial temporal regions derived from the ADHD-HC GLM analysis. Unlike the main ADHD-HC model, the models for ADHD with and without hyperactivity/impulsivity, the effect of emotion dysregulation scores, and the effect of age did not reveal any relevant significant results. For a complete overview of all significant clusters, see Table 2.

**Table 2:** Significant clusters and cluster maxima for brain regions with the individual emotion > shape and the left and right amygdala psychophysiological interaction contrasts

			MNI coordinates				
	Cluster size	Anatomical structures	*X*	*Y*	*Z*	*Z* value	*p* value
ADHD-HC model							
HC							
1	40,191	OP, FuG, LOC, IFG, MFG, AMYG, HPC, vmPFC, FP	−2	52	−20	4.82	< 0.001 9
ADHD							
4	11,438	FuG, LG, Cerebellum	2	−78	−26	4.84	< 0.001
3	1785	Right LOC, FuG	46	−70	−16	4.14	< 0.001
2	1373	Left MFG, IFG	−32	12	32	4.74	< 0.001
1	796	Left AMYG, HPC	−14	0	−12	3.70	0.007
HC > ADHD							
4	311	Subcallosal Cortex, HPC, left AMYG	−2	10	−22	3.55	< 0.001
3	287	CUN, LG	4	−76	14	3.43	< 0.001
2	258	Right STG, MTG	48	−32	2	3.90	< 0.001
1	237	Left LOC, FuG	−34	−84	−26	4.05	0.002
ADHD > HC							
No significant clusters							
PPI with left amygdala and ADHD-HC model							
HC							
2	616	FP, vmPFC	−8	60	10	4.21	< 0.001
1	143	Precuneus	−2	−64	18	3.48	0.003
ADHD							
2	149	Right SFG, FP	20	34	50	3.8	0.003
1	134	Right LOC	30	−80	14	4.13	0.008
HC > ADHD							
1	163	left PHG	16	−34	−20	3.47	< 0.001
ADHD > HD							
No significant clusters							
PPI with right amygdala and ADHD-HC model							
HC							
5	662	FP, vmPFC	6	66	22	4.13	< 0.001
4	472	OP	−10	−106	2	4.11	< 0.001
3	302	Cerebellum	−18	−70	−34	3.93	< 0.001
2	161	Left LOC, MTG	48	−62	8	3.55	0.004
1	148	Right HPC	14	−40	4	3.79	0.008
ADHD							
1	131	FP	4	58	16	3.76	0.008
HC > ADHD							
4	224	Right LOC, MTG	48	−62	8	3.63	< 0.001
3	218	FP, vmPFC	0	70	6	3.62	< 0.001
2	178	OP	−30	−92	−4	3.37	< 0.001
1	173	vmPFC	2	44	−22	4.42	0.001
ADHD > HC							
1	150	Left STG	−2	−4	74	3.46	0.004

Results are presented for the ADHD-HC GLM with groups’ mean effects and between group contrasts. Testing was conducted after FWE-cluster-correction using a z-threshold of 2.3 and a significance threshold of 0.01

*AMYG* amygdala, *CUN* cuneus, *FuG* fusiform gyrus, *FP* frontal pole, *HPC* hippocampus, *IFG* inferior frontal gyrus, *LG* lingual gyrus; *LOC* lateral occipital cortex, *MFG* middle frontal gyrus, *MTG* middle temporal gyrus, *PHG* parahippocampal gyrus, *STG* superior temporal gyrus, *vmPFC* ventromedial prefrontal cortex

#### ROI analysis with left and right amygdala

To study activation differences between emotion and shape stimuli within the amygdala in more detail, an ROI analysis was performed. None of the considered covariates showed significant main or interaction effects. Accordingly, they were not entered into the final model. Using partial *F* tests, a trend towards statistical significance for the interaction effect between diagnostic status and laterality was revealed (F(1,110) = 3.120, *p* = 0.080, partial-η^2^ = 0.028). Further, a significant main effect of diagnostic status was found (F(1,110) = 4.401, *p* = 0.038, partial- η^2^ = 0.038).

**Fig. 3:**
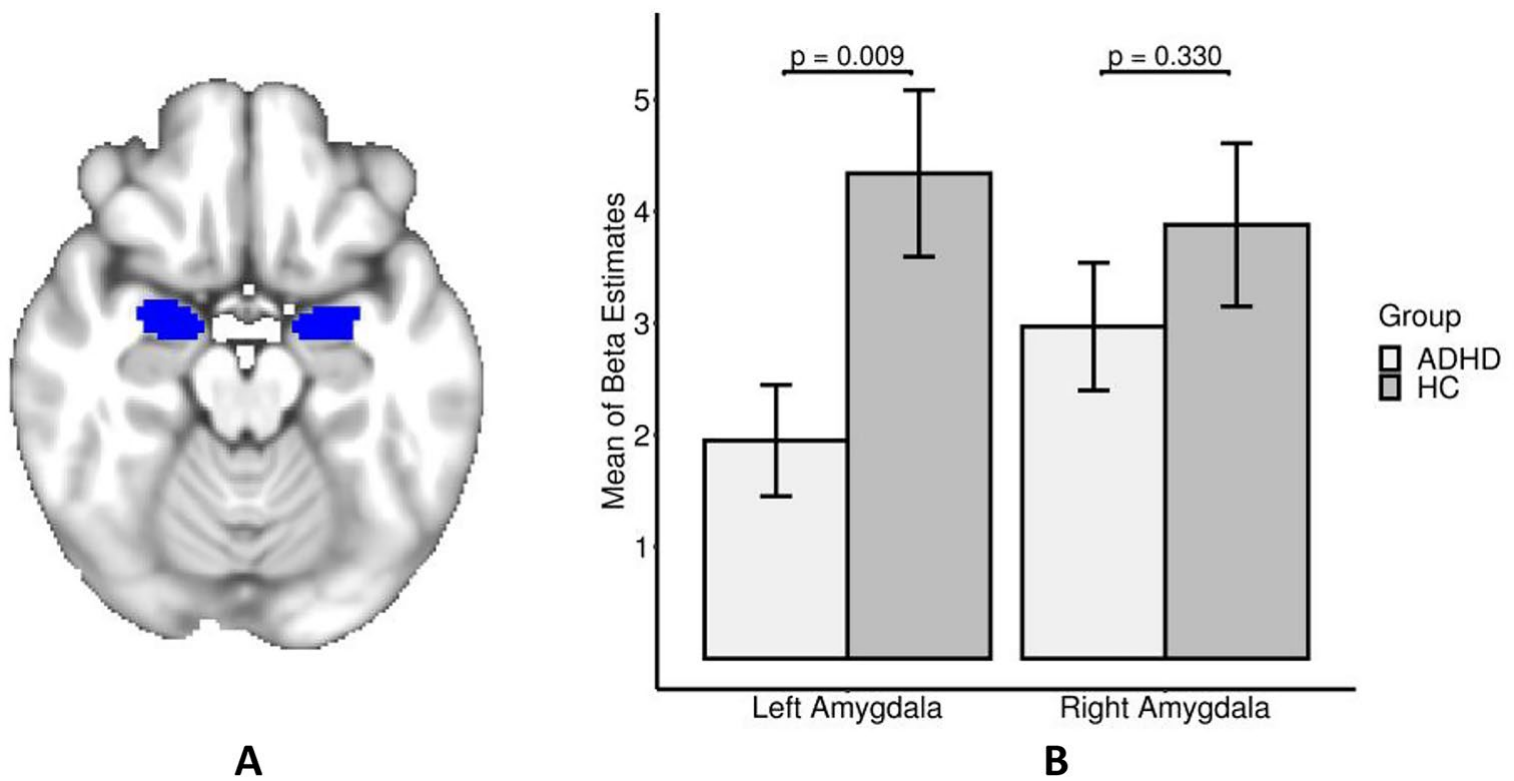
(**a**) Left and right amygdala masks that were used for ROI analysis. (**b**) Barplot with means and standard errors of beta contrast values for the different participant groups for the left and right amygdala. *ADHD* attention-deficit/hyperactivity disorder, *HC* healthy controls

Participants with ADHD had smaller activity differences in both the right and left amygdala when compared to healthy controls (Fig. 3A; left amygdala: ADHD mean ± SD = 1.95 ± 3.89, healthy control mean ± SD = 4.34 ± 5.32; right amygdala: ADHD mean ± SD = 2.97 ± 4.46, healthy control mean ± SD = 3.88 ± 5.21). Using post hoc *t* tests, only the mean difference of the left amygdala proved to be significant (*t*(df = 90) = 2.676, *p* value = 0.009, *d* = 0.522). Analyses of ADHD type, emotion dysregulation scores, reaction times, accuracy, and age did not reveal any significant main or interaction effects. However, we found a trend level significant interactive influence of group membership and left amygdala activity on reaction times and accuracy during the emotional trials.

#### Psychophysiological interaction analysis for group differences

**Fig. 4:**
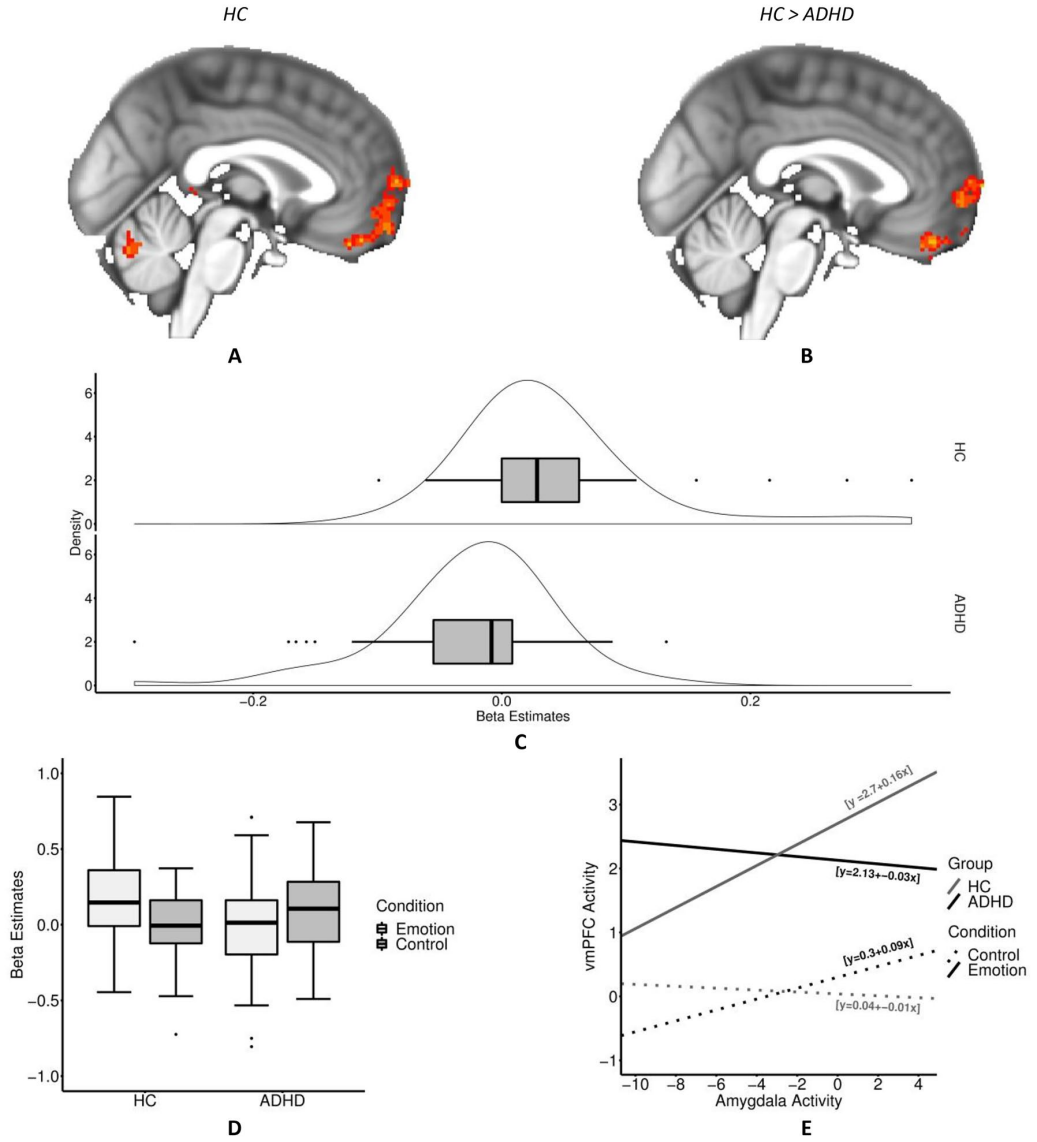
(**a**) Right amygdala × emotion > shape contrast PPI effect within the healthy control (HC) group at x-coordinate = 0. (**b**) Right amygdala × emotion > shape contrast PPI difference between the control and ADHD groups (ADHD < HC) at x-coordinate = 0. (**c**) Boxplots and underlying approximated distributions of group specific beta estimates for the PPI effect of right amygdala activity and the trial condition on vmPFC activity. (**d**) Participant group and stimuli specific boxplots of the individual slope estimates which serve as estimates for the coupling between right amygdala and vmPFC. (**e**) Participant group and stimuli specific regression lines with median intercepts and slopes that serve as estimates for the coupling between right amygdala and vmPFC

In a third step, we investigated whether task condition dependent functional connectivity between amygdala and prefrontal structures differed between healthy controls and participants with ADHD (HC > ADHD contrast). In controls, the right amygdala showed significantly stronger positive coupling with the bilateral vmPFC and frontal pole in emotional as compared to visuo-spatial trials. In contrast, levels of coupling were not significantly different between the two stimuli conditions in participants with ADHD. Here, opposing tendencies were observed. Low positive coupling between the amygdala and vmPFC and frontal pole was seen during the visuo-spatial but not the emotion condition. Statistical analysis confirmed that healthy controls showed a significantly larger vmPFC and frontal pole PPI effect than participants with ADHD (Fig. 4). The average parameter estimate for the PPI effects of the controls was 0.040 ± 0.075 and −0.026 ± 0.067 for participants with ADHD. The amplitude of average covariance change from visuo-spatial to emotional stimuli was −6.729 for controls and −3.086 for ADHD. Changes occurred in opposite directions (i.e., from negative to positive in controls and from positive to negative in individuals with ADHD). While the distribution of PPI estimates for participants with ADHD was predominantly negative and showed slight negative skewness, the distribution of controls proved to be mainly positive and showed slight positive skewness. A complete overview of all significant PPI clusters can be found in Table 2.

## Discussion

We investigated neural processing patterns of 112 adolescents/young adults with ADHD and healthy controls during the performance of an emotional face-matching task, and considered the influence of ADHD type, emotion dysregulation, and age. Participants with ADHD were overall slower and made more errors on emotional but not visuo-spatial control trials. During the processing of emotional faces as compared with the visuo-spatial stimuli, they showed less activity in the left amygdala and hippocampus, occipital regions, fusiform gyrus, and posterior fraction of the temporal cortex. The left amygdala finding was supported with a subsequent ROI analysis. Furthermore, healthy controls showed positive coupling between the right amygdala and vmPFC during emotional but not visuo-spatial trials. On the contrary, participants with ADHD showed negative coupling between the two structures during emotional trials but positive coupling during the visuo-spatial trials. For the ADHD participants, however, these task condition-specific differences were not significant.

Behavioral findings are in line with studies in pediatric and adult ADHD samples in which evidence for a reduced accuracy and delayed responding to emotional content, such as emotional faces or social feedback, was found [11–14, 51]. Our results may be partly due to a generally higher degree of task difficulty in emotion recognition compared to visuo-spatial trials (matching of finely detailed features versus simple spatial orientations). Earlier theories have suggested that emotion recognition deficits in ADHD depend on general attention deficits [52]. Alternatively, the present as well as prior findings may also indicate specific difficulties in the processing of emotional expressions of faces. Indeed, affective and social problems in ADHD may arise from the failure to specifically attend to or process the appropriate emotional cues.

The notion of deficient emotion recognition capabilities in ADHD was, to some extent, supported by the whole-brain and ROI group comparison of the task fMRI data. While the activity differences between participants with ADHD and healthy controls in structures commonly associated with general attentional performance were primarily not significant, decreased amygdala activation was found in participants with ADHD. In addition to its central importance for general affective arousal, the amygdala is indispensable for the recognition of emotions [53]. In connection with the worse behavioral results, the reduced left amygdala activity might indicate its relevance for deficient emotion recognition in ADHD. Previous studies, however, also found increases of left amygdala reactivity during tasks requiring emotional processing [15, 16] and/or only significant results for sample subgroups, e.g., only adults or children, or only in those with certain comorbidities [6]. Thus, functional alterations in relevant structures may not be homogeneous in the ADHD population, and may be task specific. Also, amygdala asymmetry in the processing of emotional stimuli was repeatedly shown [15, 17, 18]; while the left amygdala may be more involved in the analysis of local, fine-grained details, the right amygdala may be more biased towards global stimuli aspects [47].

Results of the PPI analysis further suggest that not only altered amygdala activation, but also the functional connections with medial prefrontal structures may be associated with emotion recognition in ADHD. In contrast to the healthy controls, the coupling of the right amygdala and vmPFC in participants with ADHD did not significantly depend on the emotional magnitude of the stimuli. Reciprocal connections between amygdala and vmPFC and further information relay to dorsal prefrontal structures are seen as being crucial for emotion recognition and categorization [26, 29, 54, 55]. The coupling pattern of the ADHD group, not significantly depending on the emotional content, may indicate a dysfunctional, unspecific relay of reinforcement expectation information. This is in line with previous research, which suggests that deficits in the vmPFC may hinder the integration of perceptual structures and structures that provide somatic markers for emotion recognition [56]. The reason for the asymmetry in the PPI findings may further lie in the differential roles of the vmPFC. Research indicates that the left vmPFC is more involved in reappraisal and positive emotion processing, while the right vmPFC appears to be more associated with avoidance behavior and negative emotions (as depicted by the present task’s stimuli) [57–60].

Neither behavioral results nor neural activity during task processing were related to the emotion dysregulation scores. While this may imply that processes that cause deficient emotion regulation in ADHD are not properly covered by the applied emotion matching task, it is also possible that the validity of the emotion dysregulation symptom scores, which were derived from a parent questionnaire (CPRS-R:L), is insufficient. It was recently shown that cognitive tasks and questionnaires, which are both commonly used to measure self-regulation, frequently lack an empirical relationship, while cognitive tasks only show limited ecological validity [61]. Additionally, it must be considered that the CPRS-R:L is intended for individuals up to 18 years of age. We, nonetheless, decided to take the emotion lability scores of the CPRS-R:L as the alternative would have been to combine scores from different questionnaires answered by different individuals (participant, parent, or teacher). However, our approach may have limited the validity of the scores for individuals older than 18 years. Future studies may benefit from utilizing alternative measures for emotion dysregulation since participants with ADHD did not show pronounced emotion dysregulation problems, whereas controls showed low variance in these scores.

Further, the applied task only required matching of a restricted range of emotional facial expressions without having to explicitly recognize them. Future investigations might utilize alternative experimental tasks that better capture whether a certain emotion has actually been recognized [62, 63]. It is possible that brain activity during individual trials is not limited to the recognition of emotions, as the trial length was longer than the time typically needed for emotion recognition. Particularly with regard to the observed activity and connectivity deviations of the amygdala, it cannot be excluded that those deviations are also due to attention problems. Amygdala activity is sensitive to deviant attention allocation and in relation to individuals with autism [64], for example, it has been shown that failure to pay attention to certain characteristics can have significant effects. Furthermore, it must be acknowledged that the task had relatively few trials, which may have affected the power and reliability of the connectivity analyses. This constraint also required us to conduct a block-model evaluation of the task. Since stimuli of both genders are present within the individual blocks, it was not possible to analyze the impact of the face-stimuli’s gender on the outcome measures. Additionally, the sample sizes may have been too small to detect significant differences between the different ADHD type groups. This, however, may also be due to the fact that in individuals with ADHD, the frequency of hyperactivity/impulsivity symptoms often decreases as they grow up, whereas the opposite trend is observed in emotion dysregulation [6]. Finally, with regard to control subjects, it can be noted that for several phenotypic variables they obtained above average results, which could limit their representativeness [65].

In conclusion, the current study shows a possible link between emotion recognition deficits and ADHD in adolescents and young adults. Results showed smaller BOLD-response differences between emotion and visuo-spatial trials, particularly in the area of the left amygdala, and dysfunctional connectivity between the right amygdala and vmPFC. The results may indicate that emotion recognition deficits in ADHD are associated with abnormalities in affective arousal structures and their functional connections to medial prefrontal areas. Participants with ADHD also had more emotion regulation problems than healthy controls, however, neither this, nor ADHD type, nor age were related to emotion recognition and associated neural processing alterations.

## Data Availability

Data can be obtained from the PI of NeuroImage, Prof. Jan Buitelaar.
